# Tree Water Use Patterns as Influenced by Phenology in a Dry Forest of Southern Ecuador

**DOI:** 10.3389/fpls.2018.00945

**Published:** 2018-07-06

**Authors:** Philipp Butz, Dirk Hölscher, Eduardo Cueva, Sophie Graefe

**Affiliations:** ^1^Tropical Silviculture and Forest Ecology, University of Göttingen, Göttingen, Germany; ^2^Nature and Culture International, Loja, Ecuador

**Keywords:** drought, leaf phenology, sap flux, soil moisture, stem succulence

## Abstract

Tropical dry forests are composed of tree species with different drought coping strategies and encompass heterogeneous site conditions. Actual water use will be controlled by soil moisture availability. In a premontane dry forest of southern Ecuador, tree water use patterns of four tree species of different phenologies were studied along an elevational gradient, in which soil moisture availability increases with altitude. Main interest was the influence of variation in soil moisture, vapor pressure deficit, species (representing phenology), elevation, and tree diameter on water use. Special emphasis was put on the stem succulent, deciduous *Ceiba trichistandra*, as high water use rates and drought coping involving stem succulence was to be expected. Tree water use rates increased linearly with diameter across species at high soil water content. However, when soil moisture declined, sap flux densities of the species responded differently. The stem succulent, deciduous *Ceiba* and other deciduous tree species reduced sap flux sensitively, whereas sap flux densities of the evergreen (broad leaved) *Capparis scabrida* were increasing. This was also reflected in diurnal hysteresis loops of sap flux vs. vapor pressure deficit (VPD) of the air. Under dry soil conditions, *Ceiba* and other deciduous tree species had much smaller areas in the hysteresis loop, whereas the area of *Capparis* was largely enhanced compared to wet conditions. The evergreen *Capparis* potentially had access to deeper soil water resources as water use patterns suggest that top soil drought was tolerated. The deciduous species followed a drought avoidance strategy by being leafless in the dry season. The stem succulent deciduous *Ceiba* flushed leaves at the end of the dry season before the rainy season began and also re-flushed early in the dry season after a rain event; however, water use rates at this occasion remained low. *Ceiba* was also ready for fast and strong response in water use when conditions were most favorable during the wet season. The study thus indicates a strong influence of species’ drought coping strategy on water use patterns in tropical dry forests.

## Introduction

Drylands cover approximately 42% of the global land surface (Sorensen, 2009). They receive low annual rainfall volumes with a pronounced seasonal distribution, and according to climate change scenarios they may further expand (Huang et al., 2016). The forest area in dryland biomes has recently been found to be significantly larger than previously estimated (Bastin et al., 2017). 48% of all dryland forest is located in the tropics and 63% of the tropical dry forest area has a tree canopy cover of ≥40% and are thus according to FAO (2001) definition considered as closed forest (Bastin et al., 2017). Due to their vast occurrence and resulting high diversity the variety of mechanisms through which species in tropical dry forests avoid or tolerate drought are partly unknown (Delzon, 2015).

In tropical dry forests and other regions with seasonal water limitation, tree species with different drought coping strategies often co-occur. Mainly based on leaf phenology they are classified as (i) deciduous species that shed their leaves at the beginning of the dry season and re-flush at the beginning of the wet season; (ii) deciduous, stem succulent species, which mainly differ from the former category by their big trunks and early leaf flush at the end of the dry season; (iii) brevi-deciduous tree species with a short leafless period of a couple of weeks during the dry season, during which all leaves are shed and replaced by new ones; and (iv) evergreen species (Borchert, 1996). Deciduousness is interpreted as drought avoidance, whereas evergreen species are considered drought tolerant (Borchert, 1994b, 1998; Allen et al., 2017). Likewise, the approach of Schwinning and Ehleringer (2001) for plants in water limited biomes distinguishes drought avoidance and drought tolerance based on the phenology of evergreen, drought deciduous, and stem succulent deciduous species. In general, stomata of drought-deciduous species are more sensitive to changes in soil water availability than those of evergreen species, with deciduous species exhibiting greater declines in stomatal conductance than co-existing evergreen species during dry periods (Myers et al., 1997; Eamus and Prior, 2001). For the latter, water use during the dry season is explained by deep reaching roots with access to deep water reservoirs (Elliott et al., 2006).

Among trees of a given forest and irrespective of species identity, tree water use rates and tree diameter underlie a typical scaling relationship (Meinzer et al., 2004, 2005; McJannet et al., 2007), at least under favorable conditions. Tropical dry forest trees may show similar relationships but conditions will often be non-favorable, and drought response in water use among such species remains little studied. Of particular interest are stem succulent species which occur in several taxa, often reaching extraordinary diameter classes, exceeding by far co-existing species and suggesting high water use capacities. In general, stem water dynamics are known to play an important role as an intermediate source of water for transpiration (Goldstein et al., 1998; Meinzer et al., 2001); in tropical as well as temperate regions, their contribution to total transpiration (in soft- and hardwood species) ranges from 10 to 50% (Carrasco et al., 2014). In dry forests, stem water storage so far has been linked with water demands for reproduction and leaf flushing during the dry season (Reich and Borchert, 1984; Borchert, 1994a). Studies in the stem succulent, deciduous baobab trees (*Adansonia* spp.) showed that leaf flush at the end of the dry season almost entirely depended on stem water storage; which was also important for conduit safety and turgor maintenance during the wet season, but apparently was not used for maximizing stomatal opening (Chapotin et al., 2006a,b). Astonishingly, despite tree sizes and very large diameters, the water use rates were not extraordinarily high. This indicates a rather limited role of stored stem water in baobabs for daily transpiration in the wet season; instead it enables leaf flush in the late dry season under arid conditions (Chapotin et al., 2006a,b). If this phenomenon is uniquely found in the stem succulent Baobabs of Madagascar, or as well in other species in similar biomes, remains unknown.

The present study was conducted in southern Ecuador at the western fringes of the Andes in a relatively well-preserved remnant of the Tumbesian dry forest. In this premontane tropical dry forest four tree species were studied along an elevational gradient, in which soil moisture availability increases with altitude due to higher influence of wet air coming from the Pacific. The overall objective was to analyze the importance of soil moisture changes for tree water use with respect to drought coping strategies among four phenologically different tree species (deciduous stem succulent, deciduous, brevi-deciduous, and evergreen). Next to soil water content and species, we also examined the influence of vapor pressure deficit, elevation, and tree diameter to assess particular influences of these factors in this tropical dry forest area. Of special interest was the scaling relationship of water use – diameter and phenology of the stem succulent *Ceiba trichistandra* due to its outstanding diameter and obvious water storing capacities.

## Materials and Methods

### Study Area

The study was conducted in the Laipuna Reserve (4°22′S, 79°90′W; 1.600 ha) located in the core of UNESCO “Bosque Seco” Biosphere Reserve in the Catamayo River canyon in southern Ecuador, which consists of a protected forest at altitudes between 600 and 1450 m asl. At low altitude, annual rainfall averages 540 mm with a considerable year-to-year variability (350–800 mm y^-1^ between 2007 and 2014) (Supplementary Figure S1) (Butz et al., 2017). The dry season lasts from mid-May to end of December. Also in the wet season dry spells are frequent. Higher altitudes receive an enhanced moisture input, partly from fog, due to moist air masses coming from the Pacific, and thus soil moisture increases with altitude. Annual mean temperature (at low altitude) is 23.7°C with little monthly variation (Pucha-Cofrep et al., 2015). Soils were classified as Cambisols with a high skeleton fraction (55 %). Mean slope angle is 18° and aspect is north. The forest of the Laipuna Reserve is a closed forest (tree canopy cover >40%), has an average stem density of 410 stems ha^-1^ and basal area of 28.5 m^2^ ha^-1^. At the lowest altitude stem density (320 stems ha-1) was lower than at the two higher altitudes (400 and 600 stems ha^-1^ at 860 and 1100 m asl, respectively). Thirty nine tree species were found in this forest, of which seven are evergreen and the remaining deciduous. The percentage of evergreen trees increased with altitude (Homeier, unpublished). The forest was described as premontane deciduous dry forest (Sierra, 1999; Aguirre et al., 2006).

### Studied Tree Species

Four tree species were selected for this study: *Ceiba trichistandra, Eriotheca ruizii, Erythrina velutina*, and *Capparis scabrida* (**Table 1**). *Ceiba* is a leaf deciduous stem succulent species, *Eriotheca* is deciduous, and *Capparis* is an evergreen species. *Erythrina* has been found to be brevi-deciduous according to Cueva and Acarró (2011), but in our study region showed a similar leaf exchange behavior than the other deciduous species (despite its flowering during the dry season, which is a brevi-deciduous trait). Of the total stem density, *Ceiba* had a share of 4%, *Eriotheca* of 13%, *Erythrina* of 10%, and *Capparis* of 2% (Homeier, unpublished). *Eriotheca* and *Erythrina* were observed to flush leaves with the onset of the wet season at the end of January, *Ceiba* approximately one month earlier (Supplementary Figure S2). Leaf shedding in the three deciduous species usually occurs simultaneously at the end of the wet season, most frequently beginning of June. All four species have diffuse porous wood, with wood densities ranging from 0.27 (*Ceiba*, deciduous stem succulent) to 0.77 g cm^-3^ (*Capparis*, evergreen) (Zanne et al., 2009; **Table 1**).

**Table 1:** Characteristics of the four studied tree species (mean ± SD, *n* = 4 trees per elevation), wood density taken from Zanne et al. (2009).

Species	Family	Leaf phenology/ succulence	Foliation during study period	Wood density (g cm^-3^)	DBH (cm)			Tree height (m)		
					670 m	860 m	1100 m	670 m	860 m	1100 m
*Ceiba trichistandra*	Malvaceae	Deciduous/stem succulent	Dec–Jun	0.26	65.3 ± 2.3	69.9 ± 3.1	99.6 ± 4.3	16.9 ± 3.3	17.7 ± 3.1	18.9 ± 4.3
*Eriotheca ruizii*	Malvaceae	Deciduous	Jan–Jun	0.47	39.4 ± 2.1	34 ± 2.6	44.4 ± 3.3	13.4 ± 2.3	10.7 ± 2.4	16.1 ± 4.6
*Erythrina velutina*	Leguminosae	Brevi- deciduous	Jan–Jun	0.2	32.5 ± 3.1	28.6 ± 3.7	23.5 ± 2.1	13.2 ± 1.9	8.7 ± 2	7.8 ± 1.3
*Capparis scabrida*	Capparaceae	Evergreen	All year	0.77	21.5 ± 2.6	18.7 ± 2.3	n.a.	8.9 ± 1.6	8.4 ± 2.1	n.a.

### Sap Flux

Sap flux density was measured between April 2014 and June 2015 on 44 trees in total. Four trees of three species (*Ceiba, Eriotheca*, and *Erythrina*) were assessed at an elevation of 670 m, 860 m, and 1100 m asl. *Capparis*, however, was not found within reach and in sufficient number at the highest altitude, and was therefore only assessed at the low and mid elevation. Crowns of all study trees were exposed to full sunlight; stems were bigger than 15 cm in diameter (**Table 1**). At each altitude, the study trees were located at a maximum distance of 20 m from a central logger box. Thermal dissipation probes (TDP) after Granier (1987) were used, which consisted of a pair of probes with a diameter of 2 mm and a length of 20 mm. Every tree was equipped with two sensors (one facing north, one facing south) at a height of 1.3 m above ground in the outermost xylem. Sensors were covered by Styrofoam boxes and reflective foil for protection. The upper probe of the sensor pair was continuously heated through a heating wire that was supplied with a constant power source of 120 mA. The temperature difference between the two probes was measured every 30 s and averages were stored every 30 min (CR1000 data logger and AM 16/32 Multiplexer, Campbell Scientific, Inc., Logan, UT, United States). Temperature differences were converted to sap flux density (J_s,_ g cm^-2^ h^-1^) per unit sap wood according to the original equation of Granier (1987). To assure the sap flux probes were installed fully within the high conducting sap wood (to avoid underestimation of flux according to Clearwater et al., 1999), radial sap flux profiles using heat field deformation (HFD) sensors were created.

### Radial Sap Flux Profiles and Whole-Tree Water Use

Heat field deformation sensors (ICT International, Australia) were used to assess radial variation in sap flux density. An HFD sensor consisted of three needles each with eight measurement points spaced 1 cm apart from each other and one continuous heating element. The first needle was installed above (axial direction), the second besides (tangential direction), and the third underneath (axial direction) the heating element. A styrofoam spacer of 1-cm thickness was installed between the sensor needles and the tree, to assure that the first measurement point of the sensor’s needle was directly located underneath the bark. Data was recorded every 30 s and averages stored every 30 min. Six devices were used on the three species *Ceiba, Eriotheca*, and *Erythrina* at all altitudes and were shifted every three weeks, allowing each tree with TDP sensors to be monitored. Measurements were running during the whole wet season. HFD measurements failed in *Capparis*, for which, as a simple approximation *Erythrina* profiles were adopted (due to closest resemblances in crown structure, DBH, and height). The profile data of mean daily sap flux density from the wet season was normalized to the depth of one centimeter to make it comparable to the data of the TDP method. These normalized values were fitted to a Gaussian function as this showed the best correlation to later calculate the volume of a rotated geometric solid (Oishi et al., 2008). Following this approach, radial changes in sap flux density were considered. The daily water use (kg day^-1^) of the sample trees was calculated by multiplying the TDP-derived daily sums of sap flux densities with the sap conducting area. The water conductive area for each tree was derived from tree diameter and the effective area of highly conducting sap wood recorded by HFD sensors. To derive individual tree water use rates, the respective water conducting areas were multiplied by the summed daily sap flux densities.

### Soil Water Content and Air Humidity

Water content reflectometers (CS616, Campbell Scientific, Inc., Logan, UT, United States) were used to measure volumetric soil water content (SWC, %) continuously at the three altitudes of sap flux measurements. Four probes were installed in a square of 4 m × 4 m vertically at a depth of 0–30 cm. Air temperature (°C) and relative humidity (%) were measured at open conditions near the sap flux measurements at 860 and 1100 m asl (CS215, Campbell Scientific, Inc., Logan, UT, United States) and used to calculate vapor pressure deficit of the air (VPD, hPa). Air humidity data for the lowest study altitude had to be adopted from the mid elevation, as the actual measurements at 590 m asl failed. Data was recorded every 30 s, averaged over 30 min, and stored in a CR1000 data logger.

### Data Analysis and Statistics

Interactions of daily sap flux density with SWC, VPD, species, and altitude as fixed factors, tree size (DBH) as covariate, and individual tree ID (ID) as random effect were assessed by a mixed linear model (Model 1). The following two way interactions were included in the model: SWC:VPD, SWC:species, SWC:elevation, VPD:species, VPD:elevation and species:elevation. Assumption of normal distribution and homoscedasticity were tested and fulfilled. Relationships between sap flux density, diameter, and SWC were analyzed with linear regression, the relationship of sap flux density and VPD by logarithmic regression. For analyzing tree responses during dry and wet spells within the wet season (**Figures 4–6**), the respective period was split: a two week dry period was defined with soil water content <15%, and a two week wet period with soil water content >25%. Testing of significant differences between altitudes for monthly means of SWC was done by ANOVA with Tukey test in **Table 2**, likewise for maximum sap flux densities in **Table 3**. Diurnal hysteresis loops were used to display the relationship between sap flux density and VPD under wet and dry soil conditions. All statistical analyses were conducted with RStudio version 3.4.0 (R Development Core Team, 2008). For the analysis of the mixed linear models, the lme4 R package, version 1.15 by Bates et al. (2015) was used, for visualization of least square means of SWC and VPD trends the lsmeans R package, version 2.27 - 61 and for the fitted responses the sjPlot R package, version 2.4.1. The *R*^2^ for the mixed effect model was calculated according to Nakagawa and Schielzeth (2012) with the MuMln R package, version 1.40.4.

**Table 2:** Soil water content (SWC, %) in the study period at three altitudes.

Month	670 m asl			860 m asl			1100 m asl		
	Min	Mean	Max	Min	Mean	Max	Min	Mean	Max
Dec-14	5.1 ± .9	5.5 ± 2.2^aA^	5.9 ± 1.1	4.5 ± 1.9	5.7 ± 2.1^aA^	6.1 ± 1.5	8.5 ± 2.1	9.5 ± 2.2^bA^	12.3 ± 3
Jan-15	5.4 ± 1.2	6.2 ± 3.4^aA^	15.7 ± 1.4	7.6 ± 1	9.2 ± 3.9^aB^	18.9 ± 2.6	14.5 ± 2	18 ± 4.5^bB^	30.8 ± 4.5
Feb-15	5.2 ± 2	8.8 ± 3.9^aA^	18.6 ± 5.5	7.3 ± 1.9	14.1 ± 4.9^abB^	22.7 ± 5	14.4 ± 2.7	23.9 ± 5.9^bB^	34.1 ± 5.6
Mar-15	5.9 ± .7	19.1 ± 4.6^aB^	31.9 ± 4.9	8.6 ± 1.2	24.7 ± 5.6^bC^	36.1 ± 4.3	14.3 ± 3.1	33.9 ± 5.9^cC^	40.2 ± 5.8
Apr-15	16.4 ± 2.5	23.9 ± 4.7^aB^	30.5 ± 4.2	20.1 ± 3	25.9 ± 5.3^abC^	35.4 ± 4.9	25.9 ± 4.2	33.7 ± 3.9^bC^	40.3 ± 6
May-15	6.1 ± 1.1	9.8 ± 3.7^aAB^	23.8 ± 2.9	10.4 ± 1.1	12.3 ± 4.4^aBC^	28.6 ± 3.7	14.6 ± 2.3	20.3 ± 3.9^bB^	32.3 ± 4.5
Jun-15	9.3 ± 1.5	12.1 ± 3.2^aAB^	18.5 ± 2.8	14.3 ± 2	15.1 ± 3.8^aB^	22.9 ± 3.4	25 ± 4.7	27.52 ± 2.3^bB^	32.5 ± 5.8

*n = 4 reflectometer probes at 0–30 cm depth, mean ± SD. Monthly means were tested across altitudes and across month with ANOVA followed by Tukey test. The lower case letters indicate statistical differences (*p* < 0.05) between altitudes, the upper case letters between months.*

**Table 3:** Maximum sap flux densities under dry and wet soil conditions.

	J_smax_ (g cm^-2^ h^-1^)					
Species	670 m asl		860 m asl		1100 m asl	
	SWC < 15	SWC > 25	SWC < 15	SWC > 25	SWC < 15	SWC > 25
*Ceiba*	5.8 ± 2.3^a^	15 ± 4.5^b^	5.5 ± 1.5^a^	10.7 ± 3.8^b^	12.7 ± 5^b^	14.8 ± 6.1^b^
*Eriotheca*	5.8 ± 3^a.^	7.8 ± 4.1^a^	4.8 ± 1.6^a^	15.9 ± 5.2^b^	7.3 ± 2.9^a^	9.9 ± 4.8^b^
*Erythrina*	3.1 ± 2^a^	12 ± 3.7^b^	3.8 ± 1.8	15 ± 5.6^b^	6.3 ± 2.1^a^	12.2 ± 5.7^b^
*Capparis*	15.3 ± 4.8^b^	13.3 ± 5.7^b^	12.2 ± 2.6^b^	10.1 ± 3^b^	n.a.	n.a.

*Two week period in the 2015 rain period with low (<15%) and high (>25%) soil water content (SWC). *n* = 4 trees per species, mean ± SD. Maximum sap flux densities were tested using ANOVA followed by Tukey test. Letters indicate significant difference (*p* < 0.05) between SWC, altitude, and species.*

## Results

### Soil Water Content

**FIGURE 1:**
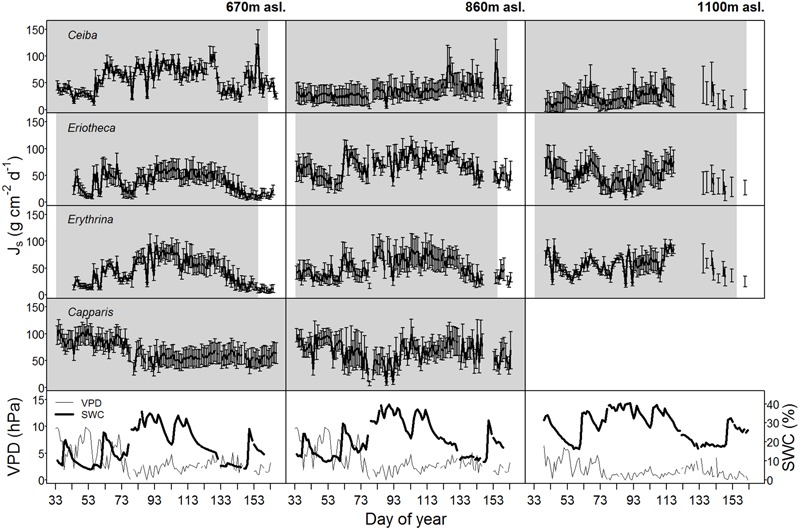
Daily sap flux densities (Js) (*n* = 4 trees per elevation) of the four study species as well as vapor pressure deficit (VPD, values at 670 m were adopted from 860 m asl) and soil water content (SWC) over the whole study period in the year 2015. Gray shaded area indicates foliated period, error bars indicate standard deviation.

Soil water content varied with season and altitude. At the mid elevation (860 m asl) lowest SWC was recorded in December 2014 at the end of the dry season (monthly mean 5.7%), and highest during the wet season in April 2015 (monthly mean 25.9%, *p* < 0.05) (**Table 2**). Along the altitudinal gradient SWC increased with increasing elevation; e.g., in March 2015 the monthly mean SWC was 19.1% at the lowest, 24.7% at the middle, and 33.9% at the highest altitude (*p* < 0.05, respectively). Also within the wet season, SWC was variable over time (**Figure 1**).

### Sap Flux in the Rainy Season

#### Species, Altitude, Tree Water Use

**FIGURE 2:**
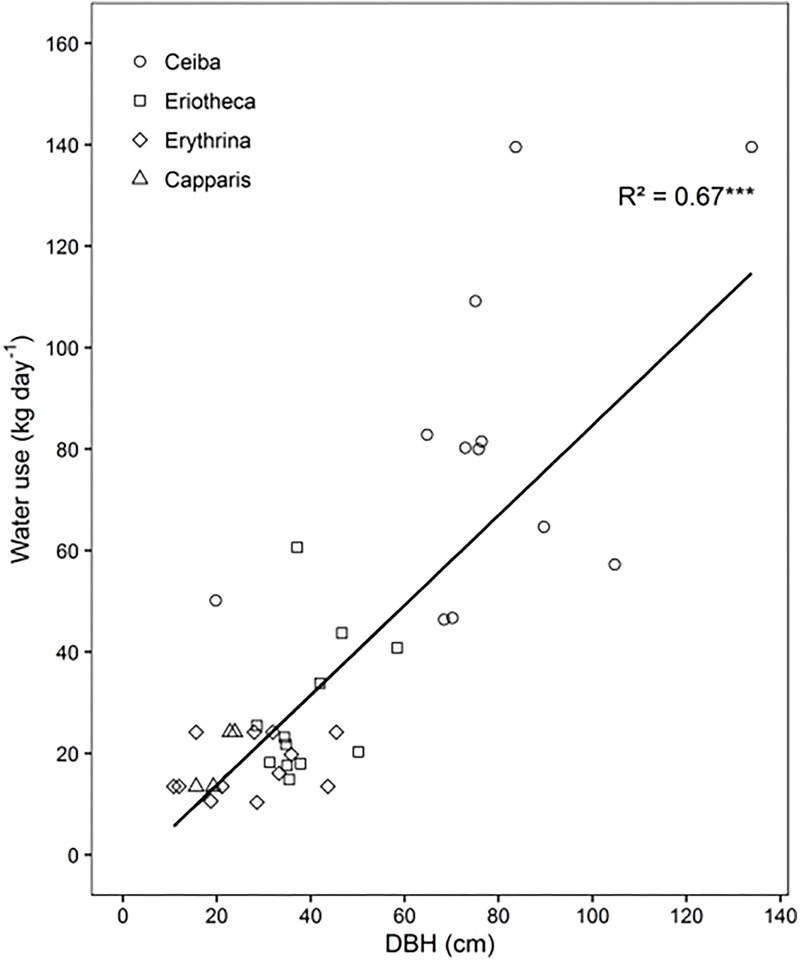
Relationship between mean daily tree water use and tree diameter (DBH), *n* = 40, *p* < 0.001.

Maximal sap flux densities (Jsmax) in the outer xylem varied from 3.1 g cm^-2^ h^-1^ (*Erythrina* at low altitude and dry soil) to 15.9 g cm^-2^ h^-1^ (*Eriotheca* at middle altitude and moist soil) (**Table 3**). Characteristic sap flux patterns under moist soil conditions on sunny days were similar between species and differed solely in magnitude (Supplementary Figure S3). At the same altitude and between dry and wet soil, Jsmax of *Ceiba, Eriotheca*, and *Erythrina* tended to be enhanced, whereas *Capparis* showed little difference. Seasonal sap flux density of all species followed fluctuations of the two main environmental drivers SWC and VPD (**Figure 1**). Radial variation in sap flux density of *Ceiba* displayed a humped-shaped curve with maximum flux densities at 1–3 cm beneath cambium and lower values at the outermost xylem (Supplementary Figure S4). *Eriotheca* in contrast had highest flux densities directly underneath the bark in the outer most xylem, whereas for *Erythrina* highest flux densities were recorded at a depth of 1 cm. On sunny days at high soil moisture, whole-tree water use increased linearly with increasing tree diameter (*R*^2^ = 0.67, *p* < 0.001) (**Figure 2**), displaying a clear scaling relationship for all species. Highest water use of 140 kg day^-1^ was estimated for a *Ceiba* tree with a diameter at breast height of 133 cm.

**FIGURE 3:**
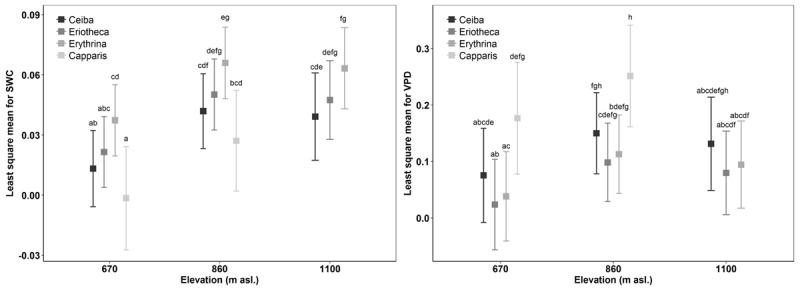
Comparison of sap flux density response to SWC and VPD for species over elevation to measure sap flux sensitivity in accordance with environmental drivers. Boxes indicate least-squares means; error bars indicate 95% confidence interval of the least square means. Means sharing a letter are not significantly different (Tukey-adjusted comparison).

The results of the ANOVA from Model 1 (interactions of the response variable sap flux density with the independent variables SWC, VPD, species, altitude, and DBH) showed significant relationships for daily sap flux density and VPD (*p* < 0.0001), species (*p* < 0.05), elevation (*p* < 0.0001), and DBH (*p* < 0.05) (**Table 4**). Significance was also found for the following two-way interactions: SWC:VPD (*p* < 0.0001), SWC:species (*p* < 0.0001), SWC:elevation (*p* < 0.0001), VPD:elevation (*p* < 0.001) and VPD:species (*p* < 0.0001). Overall the model explained 39% of the marginal variance (*R*^2^ = 0.39, fixed effects only), and 56% of the conditional variance (*R*^2^ = 0.56, including the random effect). The visualization of the fitted responses of the fixed effects from Model 1 proved the strong influence of the environmental drivers (SWC and VPD) on sap flux density (Supplementary Figure S5). The impact differed between species and altitude. *Capparis* showed low sap flux sensitivity to SWC, but the highest sensitivity to VPD (**Figure 3**). This was reflected in decreasing sap flux density with increasing SWC in contrast to a more intense VPD response (Supplementary Figure S5). The two deciduous species (*Eriotheca, Erythrina*) showed the contrary reaction patterns, indicating a stronger control of SWC than VPD. Stem succulent *Ceiba* showed for both, SWC and VPD similar sensitivity, making it the most moderate species in the setup (**Figure 3**). Under generally higher SWC at the highest plot, *Ceiba* and *Eriotheca* were able to increase sap flux density with increasing VPD (Supplementary Figure S4).

**Table 4:** ANOVA table from mixed linear Model 1 (mixed effects model) with main effects of soil water content (SWC), vapor pressure deficit (VPD), species, elevation as fixed effects, tree size (DBH) as covariate, and species identity as random effect on daily sap flux density for the whole study period.

	Sum Sq	Mean Sq	NumDF	DenDF	*F* value	Pr( > F)	
SWC	0.7747	0.7747	1	985.89	3.289	0.070039	.
VPD	6.2923	6.2923	1	984.39	26.715	2.86E-07	^∗∗∗^
Species	2.0102	0.6701	3	160.46	2.845	0.039464	^∗^
Elevation	6.949	3.4745	2	248.78	14.751	8.83E-07	^∗∗∗^
DBH	1.637	1.637	1	27.06	6.95	0.013712	^∗^
SWC:species	5.5326	1.8442	3	986.25	7.83	3.62E-05	^∗∗∗^
SWC:elevation	6.4869	3.2435	2	986.21	13.77	1.26E-06	^∗∗∗^
species:elevation	1.9887	0.3977	5	27.23	1.689	0.17104	
VPD:species	6.4311	2.1437	3	984.12	9.101	6.04E-06	^∗∗∗^
VPD:elevation	3.0612	1.5306	2	983.91	6.498	0.001571	^∗∗^
SWC:VPD	14.4382	14.4382	1	984.96	61.299	1.27E-14	^∗∗∗^

*The following two way interactions: SWC:species, SWC:elevation, species:elevation, VPD:species, VPD:elevation and SWC:VPD were included. Significance codes: ^∗∗∗^*p* < 0.0001; ^∗∗^*p* < 0.001; ^∗^*p* < 0.01; *p* < 0.5 ‘.’; *p* < 0.1 ‘ ’.*

#### Day-to-Day Response to Soil Moisture Variations

**FIGURE 4:**
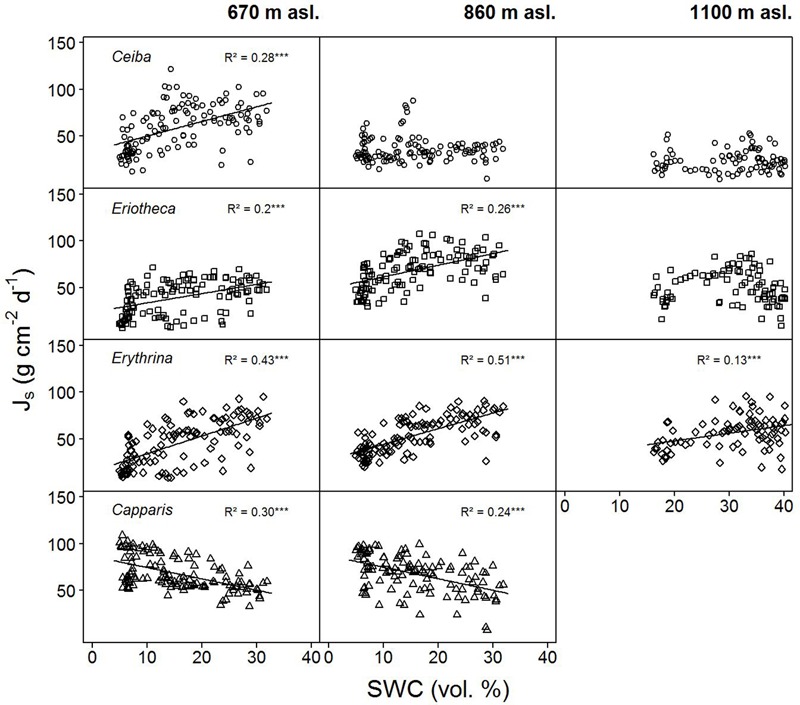
Daily sap flux densities (Js) of the four study species versus daily soil water content (SWC). Data from January to June 2015. *p* < 0.0001, ‘^∗∗∗^’, *p* < 0.001 ‘^∗∗^’, *p* < 0.01 ‘^∗^’, *p* < 0.5 ‘.’, *p* < 0.1 ‘ ’.

Sap flux density increased linearly with increasing soil moisture in the deciduous species *Ceiba, Eriotheca*, and *Erythrina*. This trend was most pronounced at the lowest elevation (*Ceiba R^2^* = 0.28, *p* < 0.001, *Eriotheca R^2^* = 0.2, *p* < 0.001 and *Erythrina R^2^* = 0.43, *p* < 0.001, **Figure 4**), and partly vanished at higher altitudes. The evergreen *Capparis* in contrast showed a linear decrease of sap flux density with increasing soil moisture at 670 and 860 m asl, this was also found for *Eriotheca* at 1100 m asl for high SWC values (> 30%). To further verify these findings, the least-squares means for SWC, species, altitude, and sap flux density (Model 1) were analyzed (**Figure 3**). Significant differences between deciduous and evergreen species in sap flux density were found for *Erythrina* and *Capparis* (*p* < 0.05) at 670 m and 860 m asl with a similar trend for the other species. *Erythrina* showed at all altitudes the highest sensitivity towards SWC. Further significant differences in sap flux density among species across altitudes were found for the study site at 670 m asl (significantly different for all species from 860 and 1100 m asl, *p* < 0.05) (**Figure 3**). This indicated a higher sensitivity of sap flux towards SWC for the deciduous species.

**FIGURE 5:**
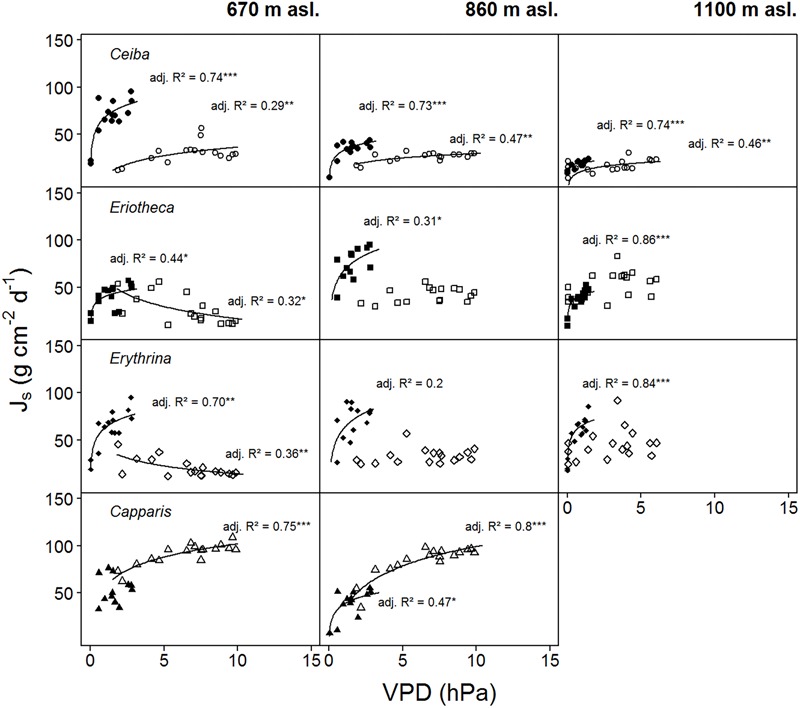
Daily sap flux densities (Js) of the four study species plotted against average daily VPD. Filled symbols indicate wet conditions (SWC > 25%), open symbols dry conditions (SWC < 15%) (data from two weeks per period). Logarithmic regressions were fitted to the data: *p* < 0.0001 ‘^∗∗∗^’, *p* < 0.001 ‘^∗∗^’, *p* < 0.01 ‘^∗^’, *p* < 0.5 ‘.’, *p* < 0.1 ‘ ’, *R*^2^ is given in italics. Daily sap flux densities derived from measurements of *n* = four individuals per species per altitude and treatment.

For two contrasting soil moisture levels (<15 %, >25 %), all deciduous species showed a decrease of sap flux density under dry conditions. *Capparis* in contrast increased sap flux density (**Table 3**). The three deciduous species increased sap flux densities with increasing VPD at moist soil conditions (**Figure 5**). Under dry conditions the increase was much less pronounced (resulting in lower *R^2^* and *p* values) or even turned into a slight decrease. At higher elevation and under dry conditions the correlations of sap flux density and VPD for *Eriotheca* and *Erythrina* disappeared, whereas for the stem succulent *Ceiba* a strong correlation was detected. As a general trend, the deciduous species displayed increasing sap flux densities with increasing VPD under wet conditions, whereas under dry conditions sap flux densities remained at low levels even with increasing VPD. However, this pattern lessened with increasing altitude. Especially under dry soil moisture levels sap flux densities of the evergreen *Capparis* showed strong positive correlations to VPD (adj. *R*^2^ = 0.75 and 0.8, *p* < 0.001, respectively at 670 and 860 m asl). Plotting the least-squares means for VPD against species and altitude for sap flux density (Model 1) over the whole study period showed a significant difference of *Eriotheca* and *Erythrina* (low sensitivity towards VPD) from *Capparis* (high sensitivity) (*p* < 0.05) at 670 and at 860 m asl (**Figure 3**). Significant difference in sap flux density within species was also recorded for all species between the altitudes of 670 and 860 m asl (*p* < 0.05) (**Figure 3**).

#### Diurnal Hysteresis at Dry and Moist Soil

**FIGURE 6:**
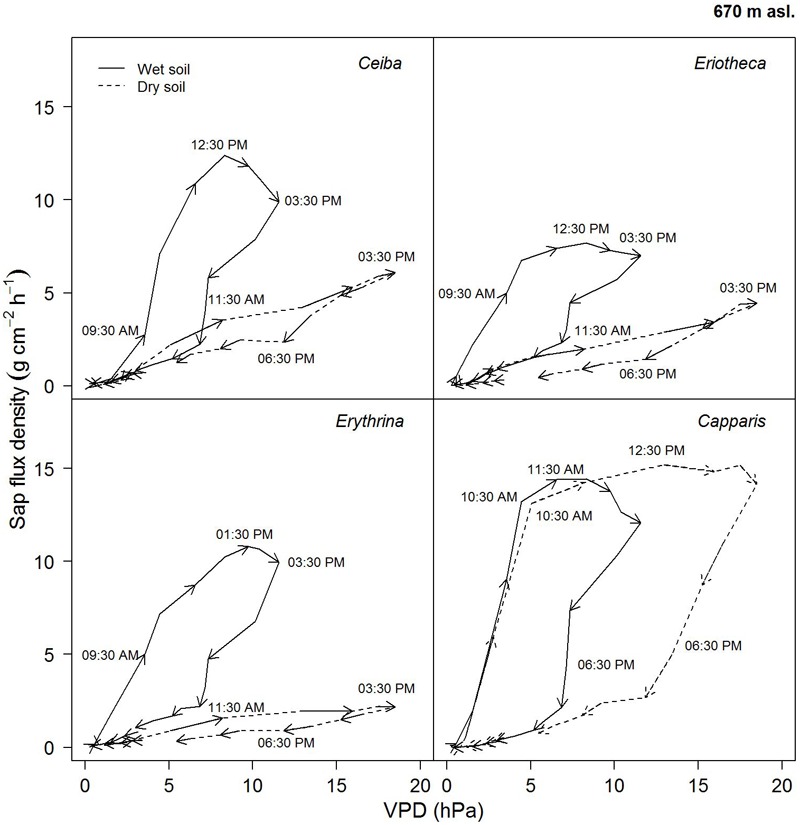
Diurnal hysteresis loops showing the relationship between hourly sap flux density and VPD for dry (16.02.2015, SWC = 7.4%) and wet (13.04.2015, SWC = 25.8%) soil conditions. Solid line indicates wet conditions, dashed line dry conditions; arrows show direction of rotation with time of the day. Hourly averages were derived from simultaneous measurement of *n* = 4 trees on a sunny day.

On a day with relatively high soil water content (26%), the four studied species showed similar diurnal relationship between sap flux density and VPD (**Figure 6**). Sap flux density rose in the morning with increasing VPD, peaked before maximum VPD around noon, and then ran back over the course of the remaining day, forming the hysteresis area. The direction of rotation was clockwise in all species. *Capparis* peaked at 11.30 am, *Ceiba* and *Eriotheca* at 12:30 pm, and *Erythrina* was the last to culminate at 13:30 pm, close to the peak of the daily VPD at 15:30 pm.

On a day with low soil water content (7 %), the area of the hysteresis loop for the evergreen *Capparis* was enhanced, whereas it was much smaller for the deciduous species. Furthermore the peaks of sap flux density shifted to 12:30 pm for the evergreen *Capparis*, and to 15:30 pm for the three deciduous species (**Figure 6**).

### Sap Flux in the Dry Period

In the dry season the evergreen species *Capparis* displayed sap flux densities comparable to the wet season, but sap flux in the defoliated deciduous species was close to zero (Supplementary Figure S6).

In 2014, some anomalies in precipitation patterns were recorded, with a low rainfall volume in April (18 mm) but 70 mm of rainfall in early May. All three deciduous species had shed their leaves by end of April; most *Eriotheca* and *Erythrina* remained defoliated until January 2015. Some *Ceiba* individuals, however, re-flushed and maximal sap flux densities of 5–8 g cm^2^ h^-1^ were measured (Supplementary Figure S7). Also some *Erythrina* trees re-flushed but sap flux data are not available.

## Discussion

The four studied tree species showed distinct patterns in water use and its response to changes in soil moisture. Maximum sap flux densities were medium to low when compared to other tree species from tropical dry forests (Kume et al., 2007; Goldstein et al., 2008; Reyes-García et al., 2012). This might be due to the fact that the other studies were characterized by higher VPDs (15 hPa vs. 30 hPa) and therefore high demanding conditions for transpiration. Under favorable conditions, daily tree water use increased with tree diameter, which has already been found in many other studies (e.g., James et al., 2003; Meinzer et al., 2005; Kunert et al., 2012), whereas under dry conditions this relation became weaker (*R*^2^ = 0.51, *p* < 0.0001, data not shown). Small statured trees including the evergreen *Capparis* had daily water use rates of less than 20 kg day^-1^, whereas the stem succulent *Ceiba* reached water use rates of about 140 kg day^-1^. Accordingly, *Ceiba* displayed a radial flux profile with the biggest area where maximum sap flux was located at 2 cm depth, protected by the outer low-conducting xylem. Temporal changes in SWC across the altitudinal gradient influenced sap flux of the species differently. The deciduous species reduced sap flux sensitively, even when in leaves, which was not observed for the evergreen species *Capparis*.

The particular response in sap flow to top soil water decrease by the evergreen *Capparis* points to access to wetter, deeper soil by deep reaching roots. Under those conditions species like *Capparis* lean towards adaptations that maximize deeper soil water use (large root:shoot ratio, predominantly deep root system, lower leaf conductance with low stomatal sensitivity; Schwinning and Ehleringer, 2001). In this context the application of stable isotopes would be an important addition to access soil water depths (Ehleringer and Dawson, 1992). Thus based on the phenology and immediate drought response *Capparis* can be classified a drought tolerant species according to the criteria described by Ogburn and Edwards (2010). The considerable reduction in sap flux density in response to soil water decrease of the deciduous (non-stem succulent) *Eriotheca* and *Erythrina* may be explained by increased stomata control. This was also reflected by the influence of VPD on sap flux, since both species had significantly lower values than the evergreen *Capparis*. Therefore both species can be classified as drought avoiding according to Ogburn and Edwards (2010).

The deciduous, stem succulent *Ceiba* had high water use rates under favorable conditions and much reduced sap flux densities when soil moisture was low. Herein the role of the big stem is of particular interest as the water storage mechanism was suspected to serve as buffer to allow for daily transpiration even under non-favorable conditions. Using dendrometer bands in a previous study, it was shown that the *Ceiba* stem decreased in circumference when the soil dried out and swiftly increased after rain events (Butz et al., 2017). For stem deciduous, stem succulent baobab trees (*Adansonia* spp.) in Madagascar, studies by Chapotin et al. (2006b) indicated that stem water storage was not used for maximizing stomatal opening and hence water use. In our study and for *Ceiba* we cannot differentiate whether water first goes into the storage and thereafter is used for transpiration or address the residence time of the water in the stem. It seems that *Ceiba* does both when water is available: increase the stem volume, most likely by increasing its water content, and transpire at high rates. We observed a reduction in sap flux accompanied by a decrease in stem circumference when soil dried out (Butz et al., 2017). Some internally stored water seemed to be used for maintaining a relatively high transpiration rate under drying soil conditions. However, transpiration was also sensitively reduced; further the strong difference between the hysteresis loops and dry and wet soil conditions underlined this strong reduction in sap flux. Only *Capparis* was able to enhance sap flux rates under drier conditions (decreased SWC, increasing VPD), indicating once more the access to deeper soil water reserves and potentially stronger stomatal control (to keep the stomata opened). The deciduous species were not able to keep sap flux rates stable. We recorded leaf flush of *Ceiba* approximately one month before the onset of the wet season under very dry soil water conditions and a subsequent decrease in stem circumference (Butz et al., 2017). Hence, there is some water stored in the stem over several months to enable leaf flushing. Chapotin et al. (2006a) suggested that baobabs use stored stem water to flush new leaves at the end of the dry season, just before the onset of the wet season. In this respect *Ceiba* and baobab seem to be similar. Also after rain events in the early dry season *Ceiba* flushed new leaves but sap flux densities remained relatively low. This contrast with results from an irrigation experiment in Costa Rica (Borchert et al., 2002) in which the deciduous, stem succulent species did not re-flush but remained dormant. The re-flush of *Ceiba* may as well be related to the fact that the intermittent rainfall event took place very early in the dry season. Trees probably were not yet dormant and thus re-flush was still under exogenous instead of endogenous control (Borchert, 1994b).

To summarize, we adopt an approach by Schwinning and Ehleringer (2001) developed for desert plants. The relation of water use to water availability in the four tree species is: (i) evergreen trees with particular response to soil drought, most likely with access to deep soil water (*Capparis*), (ii) deciduous trees with modest daily water use rates and strong reduction of sap flux in response to soil drought, but no large stem water storage (*Eriotheca* and *Erythrina*), and (iii) deciduous, stem succulent trees with high water use under favorable conditions, sensitive reduction of sap flux with soil moisture decline, responsive to short and strong rain events, and early re-flush at the end of the dry season based on water stored in the stem (Ceiba). The study thus indicates a strong influence of species’ drought coping strategy on water use patterns in tropical dry forests.

## Author Contributions

PB, DH, EC, and SG designed the study and contributed to the interpretation and discussion of results. PB conducted the experiments, analyzed the data and wrote the first draft of the manuscript. PB, DH, and SG wrote sections of the manuscript. All authors contributed to manuscript revision, and read and approved the submitted version.

## Conflict of Interest Statement

The authors declare that the research was conducted in the absence of any commercial or financial relationships that could be construed as a potential conflict of interest.
