# Supplementary material for: Tree Water Use Patterns as Influenced by Phenology in a Dry Forest of Southern Ecuador
Source: Front Plant Sci. 2018 Jul 6;9:945. doi: 10.3389/fpls.2018.00945 (PMC6043675; doi:10.3389/fpls.2018.00945)
Supplement: Supplementary file 1 [file Data_Sheet_1.docx]

Supplementary Material

**Tree water use patterns as influenced by phenology in a dry forest of southern Ecuador**

**Philipp Butz^1*^, Dirk Hölscher^1^, Eduardo Cueva^2^, Sophie Graefe^1^**

*** Correspondence:** Philipp Butz: [butzens@gmail.com](mailto:butzens@gmail.com)

*Annex*


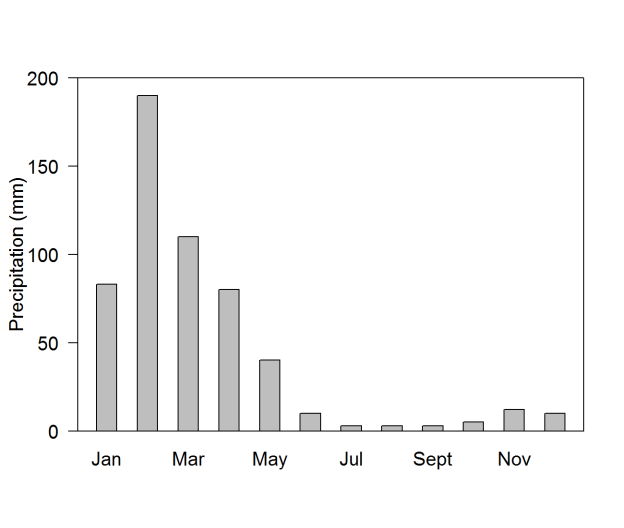


Fig.1. Monthly rainfall in the study area at 590 m asl. from 2007-2014. Mean annual precipitation was 540 mm (Butz et al. 2016).


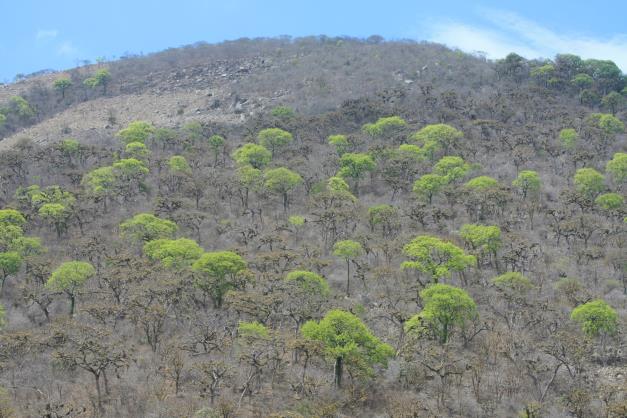


Fig.2 *Ceiba trichistandra* at the end of the dry season (December) is already flushing new foliage,

while the other deciduous species remain leafless for another month.


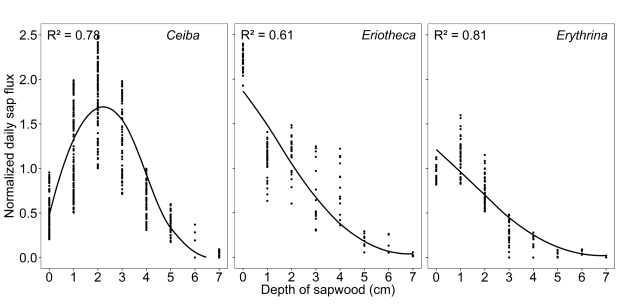
Fig.3 Radial sap flux profiles for *Ceiba*, *Eriotheca* and *Erythrina* derived by heat field deformation measurements*.* Sapwood depth starts at the cambium interface. Points indicate mean daily sap flux values per tree, normalized to the depth of 1 cm (for comparability with TDP probes), data were fitted to Gaussian equation.


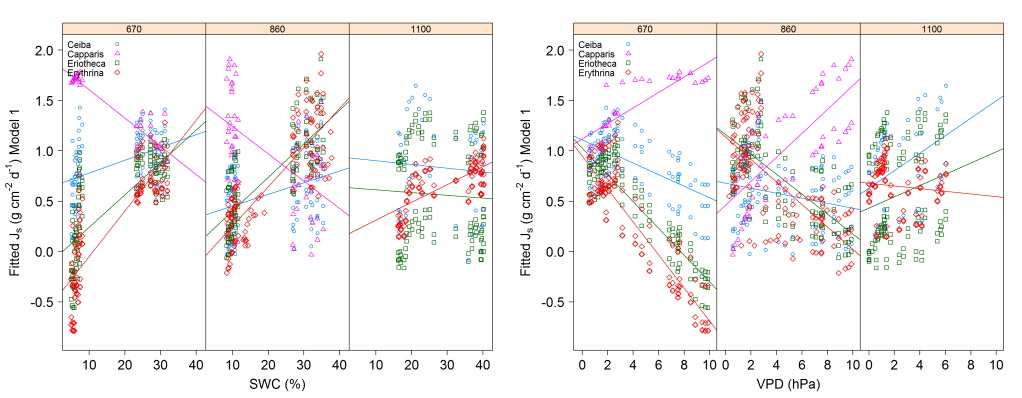
Fig.4 Fitted responses of sap flux density to SWC and VPD at 670m, 860m and 1100m asl. obtained from Model 1.


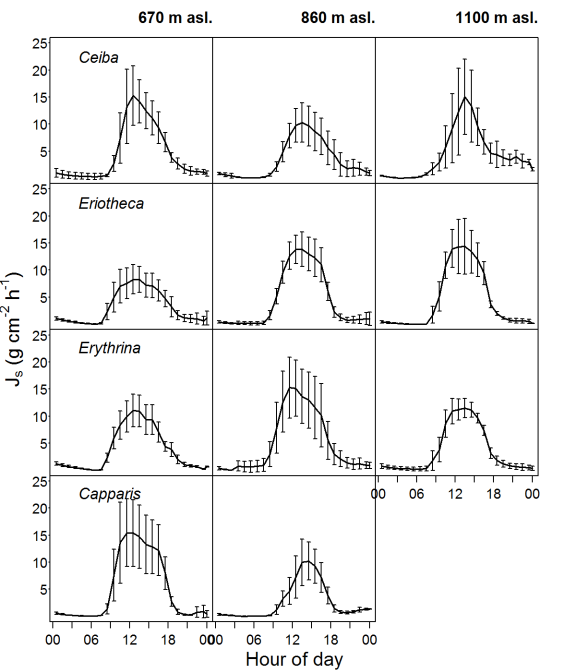


Fig.5 Characteristic daily sap flux densities (J_s_) of the four study species on a sunny day with high soil water content during the wet season at three altitudes. Means and standard deviations, n= 4 trees.


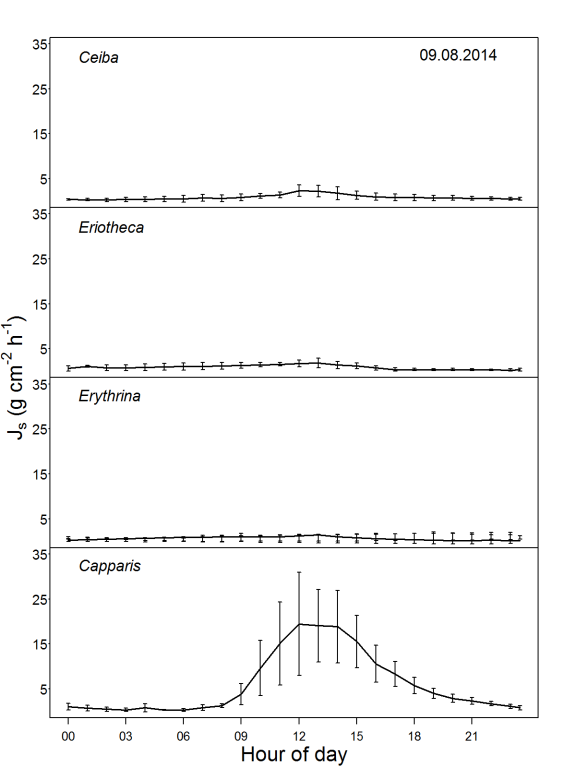


Fig.6 Characteristic daily sap flux densities (J_s_) of the four study species on a sunny day with low soil water content (< 15 %) during the dry season at 670 m asl., deciduous species were defoliated. Means and standard deviations, n= 4.


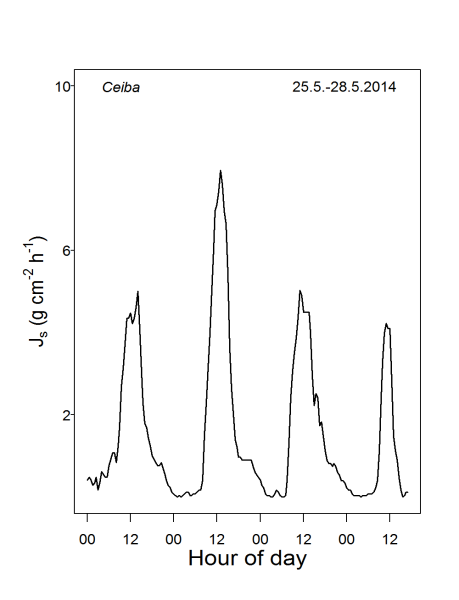


Fig.7 Characteristic daily sap flux densities (J_s_) of one *Ceiba* tree during the dry season 2014, graph shows C*eiba* for four days after an intermittent rain fall event during the early dry season after the tree re-flushed its leaves.
